# Mixed-Pattern Ameloblastoma of the Anterior Mandible: A Rare Histopathological Presentation at an Infrequent Location

**DOI:** 10.7759/cureus.42840

**Published:** 2023-08-02

**Authors:** Ourania Schoinohoriti, Christina Tsami, Vasiliki Karathanasi, Nikolaos Kolomvos

**Affiliations:** 1 Department of Oral and Maxillofacial Surgery, School of Dentistry, University of Athens, Athens, GRC; 2 Department of Dermatology, Evangelismos General Hospital, Athens, GRC

**Keywords:** resection, acanthomatous subtype, follicular subtype, mixed pattern, anterior mandible, ameloblastoma

## Abstract

Ameloblastoma is a benign odontogenic tumor of epithelial origin that exhibits a locally aggressive behavior with a high level of recurrence and multiple factors involved in its molecular pathogenesis.

This article is a case report of a 46-year-old male patient suffering from a progressively enlarging tumor of the anterior mandible that caused gradual expansion of the lingual cortical plate and root displacement without resorption of the involved teeth. Incisional biopsy was consistent with “conventional” ameloblastoma, showing a mixed pattern of both the follicular and acanthomatous subtypes. This diagnosis was corroborated through a histopathological examination of the resected specimen. The patient was submitted to en bloc resection (marginal mandibulectomy) with preservation of the lower mandibular border; dental rehabilitation was achieved through a removable prosthesis. He remains disease-free for 5.5 years postoperatively and is highly satisfied with mastication and speech.

The objective of this report is to highlight a relatively rare histopathological presentation of the “conventional” ameloblastoma, involving a site not commonly affected by ameloblastomas, the anterior mandible and crossing the midline, in a relatively young male patient.

## Introduction

Ameloblastoma is one of the most common benign odontogenic tumors, with an annual global incidence of 0.5 cases per million person-years and a widely variable geographic prevalence [1,2]. It has been reported to represent 1% of all head and neck tumors and is more commonly diagnosed between the third and fifth decades of life, with a male predilection [1].

Most ameloblastomas (approximately 70%-80%) occur in the mandible, mainly in the molar region and ascending ramus (70%), followed by the premolar (20%) and anterior region (10%); 20% of the tumors may arise in the maxilla, predominantly in the canine and molar region [1,3]. However, the rare desmoplastic subtype, representing 2% of all ameloblastomas, arises most frequently in the premolar and anterior regions of both the mandible and maxilla [2].

Although ameloblastoma usually presents clinically as a slow-growing swelling of the involved jaw region without specific symptoms, pain and disfigurement occasionally correlate with advanced size. Radiologically, ameloblastomas are described as either unilocular or multilocular osteolytic lesions, leading to cortical bone spacious thinning and expansion, with or without dental root absorption [1].

Ameloblastoma is considered a potentially aggressive, locally invasive, benign tumor that presents a high recurrence rate but usually does not metastasize. According to the 2017 WHO classification, malignant (metastasizing) ameloblastoma represents an incredibly rare variant that metastasizes mainly into the lungs [1,4].

Various histological subtypes of ameloblastomas have been described, including the most prevalent, follicular (64.9%), followed by the plexiform (13%), desmoplastic (5.2%), and acanthomatous (3.9%) variants [5]. Moreover, there are only a few reports presenting ameloblastomas of the rare acanthomatous subtype that were located at the anterior region of the mandible and crossed its midline [6-8].

The objective of this article is to report our experience with treating an interesting case of mixed-pattern ameloblastoma, including components of both the follicular and acanthomatous subtype, located at the anterior mandible and crossing the mandibular midline of a relatively young male patient.

## Case presentation

A 46-year-old male patient attended the outpatient clinic of our department, complaining of a progressive swelling at the lingual aspect of his anterior mandible, deteriorating over the last six months. The patient reported neither pain nor any other symptoms. His medical and dental histories were unremarkable.

**Figure 1:**
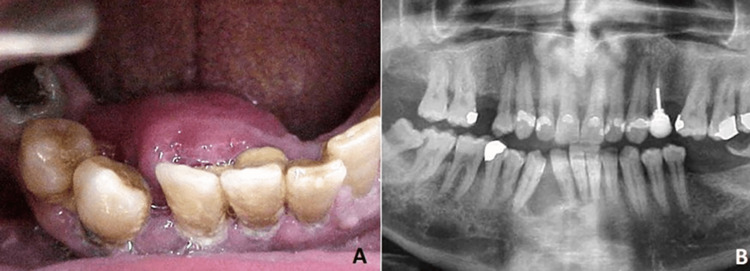
A: Intraoral photograph of the patient showing the expansion of the lingual cortical plate at the anterior mandible. B: Original orthopantomogram of the patient.

Upon clinical examination, the patient exhibited a considerably expanded lingual cortical plate at the anterior region of the mandible, extending from tooth #43 to tooth #31 along with light motility of teeth #41, #42, and #43 (Figure 1A). On palpation, the tumor was bony hard in consistency, noncompressible, and non-fluctuant. Pulp vitality tests of all involved teeth were negative (nonvital). Orthopantomogram revealed a unilocular, relatively well-defined radiolucent lesion of the symphyseal and parasymphyseal region that extended from tooth #43 to tooth #31 and had caused root displacement, but not resorption (Figure 1B).

An incisional biopsy was directly performed. The histopathological examination of the specimen confirmed the clinical diagnosis of ameloblastoma and revealed a mixed pattern, including components of both the follicular and acanthomatous subtypes.

**Figure 2:**
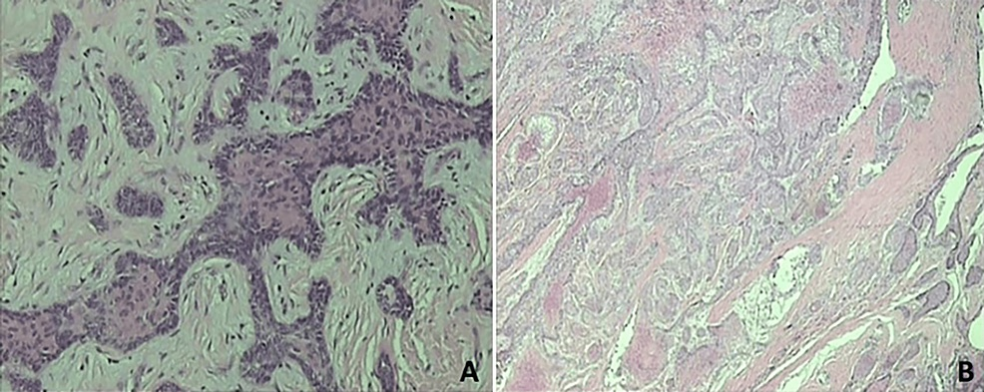
Microphotographs of the specimen following hematoxylin and eosin staining. A: The follicular component of the tumor, consisting of islands of odontogenic epithelium within fibrous stroma: peripheral tall cylindrical cells that resemble ameloblasts and surround central regions with loosely arranged epithelial cells, mimicking the asteroid network. B: The acanthomatous component of the tumor showing squamous differentiation with keratin formation within the central epithelial islands of the follicular ameloblastoma.

Based on the size and location of the lesion, the patient was submitted under local anesthesia to resection with adequate margins (marginal mandibulectomy) through an intraoral approach, along with extractions of teeth #31-#43, in an attempt to preserve as much of the healthy mandibular bone as possible. The operative site was primarily closed, and the resected specimen was sent for histopathological examination that corroborated the original diagnosis (Figure 2).

**Figure 3:**
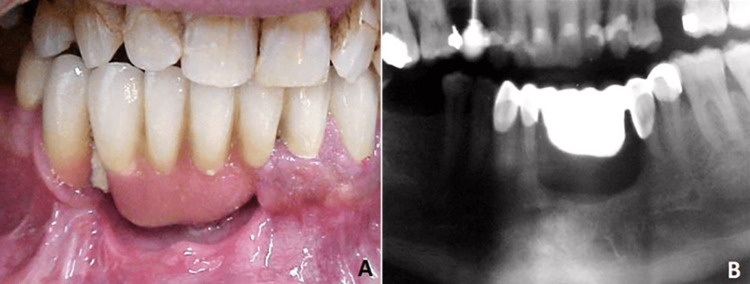
A: Intraoral photograph of the patient showing the removable prosthesis placed three months postoperatively. B: Orthopantomogram of the patient three months postoperatively.

The healing of the defect was uneventful, and a removable prosthodontic device was placed three months postoperatively to achieve the patient’s functional rehabilitation (Figure 3). The patient has been followed up for 5.5 years and remains recurrence-free and satisfied with speech and mastication.

## Discussion

According to the 2017 WHO classification, ameloblastomas are classified as “conventional” or plain ameloblastomas, unicystic, and extraosseous/peripheral. The term “solid/multicystic” included in the 2005 WHO classification to describe “conventional” ameloblastomas has been dropped as lacking biological significance and leading to confusion, while the desmoplastic ameloblastoma has been reclassified as a histological subtype and not a clinicopathologic entity [4]. Correlations between the mutational status of ameloblastoma (SMO and BRAF mutations) and specific histological subtypes and/or clinical outcomes have not been fully elucidated and should be further investigated through studies based on large, multicenter series [4].

Among the “conventional” ameloblastomas, the follicular and plexiform histological subtypes are encountered more frequently (65%-70% and 13%-14%, respectively), while the acanthomatous subtype only rarely (3%-4%) [3,5]. Moreover, the existence of multiple histopathological patterns within the same tumor upon initial diagnosis of ameloblastoma has been sparsely reported in the literature [1,5,8].

More specifically, acanthomatous metaplasia has been reported in 8%-45% of the cases and may involve a limited or greater area of the tumor. Although this alteration does not imply a more aggressive biological behavior, the tumor must be histologically distinguished from squamous cell carcinoma and acanthomatous odontogenic tumor. Preservation of the typical histological pattern of follicular ameloblastoma in certain areas of the tumor facilitates final diagnosis, while the absence of penetration at the frontal area and cell atypia allows differentiation from squamous cell carcinoma.

The acanthomatous subtype has been reported to occur mostly in the posterior mandible of older patients, with a peak incidence during the seventh decade of life (mean age: 61.3 ± 1.2 years) [5]. To our knowledge, only a few cases of acanthomatous ameloblastomas affecting the anterior mandible have been reported in the literature, either in young [6,7] or relatively elderly patients [8]. Acanthomatous metaplasia has been observed to correlate with chronic irritation, attributed to calculus and oral sepsis. Bhuyan et al. (2019) reported a case of changing histopathological pattern from plexiform type to acanthomatous type between the original diagnosis of ameloblastoma at the posterior mandible and its recurrence at the same location 17 years after treatment [9].

In our patient, who remarkably exhibited mixed-pattern ameloblastoma with components of both the follicular and acanthomatous subtypes, the tumor involved the symphyseal and parasymphyseal region of the mandible and crossed the midline. Although the possibility of originally follicular ameloblastoma undergoing acanthomatous metaplasia cannot be excluded, this seems inconsistent with the patient’s young age (46 years) and the relatively short history of the tumor (six months). However, the presence of calculus in close proximity to the tumor cannot be ignored as a local deleterious factor, potentially inducing acanthomatous metaplasia.

The treatment of “conventional” ameloblastoma has been controversial because of its unique biological behavior as a slow-growing, locally invasive tumor with a high recurrence rate. Further elucidation of the molecular factors, orchestrating its pathogenesis and recurrence, is anticipated to lead to new diagnostic markers and targeted drug therapies [10]. However, surgical treatment currently remains the gold standard for its management and may be either conservative (enucleation or excision and curettage) or radical (en bloc resection with 1-1.5 cm margins) followed by reconstruction [3]. Recurrence rates of ameloblastoma are reportedly as high as 15%-25% after radical treatment and 75%-90% after conservative treatment [3].

In our case, a “rational radical conservative” resection, consisting of marginal mandibulectomy with preservation of the lower border, was opted for to maintain the continuity of the mandible and the contour of the lower third of the face, considering the size of the tumor and its relatively well-circumscribed nature, not infiltrating the surrounding soft tissues. Thus, dental rehabilitation was achieved through a removable prosthesis that yielded an excellent functional outcome, as far as mastication and speech were concerned.

Since the recurrence rate remains persistently high even following radical resection, a close and prolonged follow-up regime should be scheduled for all patients suffering from “conventional” ameloblastomas [3]. Our patient who admittedly showed a high level of compliance remains recurrence-free for 5.5 years postoperatively.

## Conclusions

Although “conventional” ameloblastoma is a relatively common odontogenic tumor, the incidence of the acanthomatous subtype is reportedly very low. We herewith present a rare case of mixed-pattern “conventional” ameloblastoma, including a component of the acanthomatous subtype, that was located at the anterior mandible and crossed the midline in a relatively young male patient. The patient was submitted to marginal mandibulectomy and dental rehabilitation through a removable prosthodontic device and remains disease-free for 5.5 years postoperatively.
